# Complex networks analysis reinforces centrality hematological role on aerobic–anaerobic performances of the Brazilian Paralympic endurance team after altitude training

**DOI:** 10.1038/s41598-022-04823-w

**Published:** 2022-01-21

**Authors:** Fabio Leandro Breda, Fúlvia Barros Manchado-Gobatto, Filipe Antônio de Barros Sousa, Wladimir Rafael Beck, Allan Pinto, Marcelo Papoti, Pedro Paulo Menezes Scariot, Claudio Alexandre Gobatto

**Affiliations:** 1 Laboratory of Applied Sport Physiology, School of Applied Sciences, University of Campinas, Rua Pedro Zaccaria, 1.300, Jardim Santa Luíza, Limeira, São Paulo 13484-350 Brazil; 2 Laboratory of Endocrine Physiology and Physical Exercise, Department of Physiological Sciences, Federal University of São Carlos, São Carlos, SP Brazil; 3 School of Physical Education, University of Campinas, Campinas, SP Brazil; 4 Brazilian Synchrotron Light Laboratory, Brazilian Center for Research in Energy and Materials, Campinas, SP Brazil; 5 School of Physical Education and Sport of Ribeirão Preto, University of São Paulo, Ribeirão Preto, SP Brazil

**Keywords:** Computational biology and bioinformatics, Physiology

## Abstract

This study investigated the 30-days altitude training (2500 m, LHTH-live and training high) on hematological responses and aerobic–anaerobic performances parameters of high-level Paralympic athletes. Aerobic capacity was assessed by 3000 m run, and anaerobic variables (velocity, force and mechanical power) by a maximal 30-s semi-tethered running test (AO30). These assessments were carried out at low altitude before (PRE) and after LHTH (5–6 and 15–16 days, POST1 and POST2, respectively). During LHTH, hematological analyzes were performed on days 1, 12, 20 and 30. After LHTH, aerobic performance decreased 1.7% in POST1, but showed an amazing increase in POST2 (15.4 s reduction in the 3000 m test, 2.8%). Regarding anaerobic parameters, athletes showed a reduction in velocity, force and power in POST1, but velocity and power returned to their initial conditions in POST2. In addition, all participants had higher hemoglobin (Hb) values at the end of LHTH (30 days), but at POST2 these results were close to those of PRE. The centrality metrics obtained by complex networks (pondered degree, pagerank and betweenness) in the PRE and POST2 scenarios highlighted hemoglobin, hematocrit (Hct) and minimum force, velocity and power, suggesting these variables on the way to increasing endurance performance. The Jaccard’s distance metrics showed dissimilarity between the PRE and POST2 graphs, and Hb and Hct as more prominent nodes for all centrality metrics. These results indicate that adaptive process from LHTH was highlighted by the complex networks, which can help understanding the better aerobic performance at low altitude after 16 days in Paralympic athletes.

## Introduction

Hypoxia training is a powerful strategy when used correctly^1^ and can be performed in different ways, including the live high and train high (LHTH) model^2^. In this case, athletes are taken to altitude to perform part of their training program in a location with reduced partial pressure of oxygen. This proposal aims to improve sports performance, providing some advantages over opponents^1,3^. Considering the Olympic level, which differences performance among athletes are typically less than 0.5%^4^, this strategy has been conducted during annual training planning/periodization aiming improve their results. However, the positive effects of the LHTH model on physical adaptations and sports performance are dependent on different factors, such as the altitude where the training programs are conduceted^5^, exposure time and hypoxia dose^4,6^, intensity physical effort performed under the hypoxia conditions^7^ and the physical-sporting level of the athletes^5,8^. The ideal altitude for the LHTH is not yet defined, but most studies have been carried out at heights ranging from 2000 to 3000 m^9^. The literature also suggests that an exposure of at least 12 h a day for at least 3 weeks at 2100–2500m is necessary for important aerobic and performance-related adaptations^10^.

Among the main physiological effects of this process are the increase in the concentration of red blood cells and hemoglobin, responsible for transporting oxygen from the lungs to the tissues, resulting in a significant gain in aerobic conditioning and performance in medium and long-distance events^11–13^. However, changes in performance after periods of training at altitude may be related to a multifactorial etiology^14^. Inadequate training, insufficient storage of iron in the body and the presence of infections can negatively influence the hematological variables^10,15,16^, hindering the benefits of LHTH. Although hematological changes resulting from training at altitude can improve the supply and use of oxygen, the lower availability of oxygen at high altitudes can also reduce the intensity of physical stimuli applied in this environment^1,4,17^. The period of acclimatization to moderate altitude may vary among athletes, requiring an adaptation of 2 weeks so that athletes do not reduce their performance in a given competition^18^. During the altitude acclimatization process, the central nervous system, as well as the endocrine, respiratory and cardiovascular systems, undergo modifications to optimize oxygen transport, ensuring an adequate energy supply to tissues and organs^14,17,19^. As a result, performance benefits are expected to occur when returning to the lower altitude levels where testing is normally performed.

Although scientific research has also shown some negative results in terms of declining intensity in training under hypoxia, elite athletes are rarely asked to rate the intensity of training at high altitudes^20^. Some authors claim that the LHTH model is effective in improving the relative intensity of training in elite endurance athletes, contributing to increased performance. Recently, Sharma et al.^21^ evaluated a group of eight elite athletes in a 4-week phase of training at altitude (LHTH, at 2100 m) and obtained good results with a 5% increase in hemoglobin concentration and a 1.1% improvement in performance of the participants. In this proposed model, unlike the more conservative ones that point to a reduction in intensity and the need for a period of adaptation to altitude^4,9,19^, these authors managed to intensify the load/volume of training at the beginning of altitude time course.

Furthermore, from a physiological point of view, many doubts remain about the correct period of readaptation at sea level or test site after training at altitude, so that elite athletes can reach their maximum performance^22^. As far as we know, all studies investigating the effects of the LHTH model on physiological adaptations and performance have applied conventional statistical methods to interpret the data. However, despite these studies bringing important contributions, we believe that training at altitude, as well as adaptive responses to LHTH programs, are complex and deserve to be investigated in a more integrated way.

When athletes submitted to this type of intervention are already highly trained, it is possible that important physiological and/or performance changes occur, but they are not always detectable by statistical analyses based on more conventional approaches. In previous studies, we worked with more integrated models to investigate physical exercise^23,24^, and the context related to sports^24,25^, applying the concepts of complex networks^26^ for the interpretation of biological data, as discussed by some authors^27,28^. This line of research has contributed strongly to the understanding of biological responses that do not depend only on an isolated factor. Here, we believe that complex network centrality metrics can strongly help in interpreting the effects of LHTH on the aerobic, anaerobic and performance responses of elite athletes. Thus, centrality metrics, such as *pondered degree* (nodes with the highest number of connections with others in the same scenario), *pagerank*, which is used as a classifier for the most important nodes in the network, and *betweenness*, which highlights the node that represents its importance given its intermediation between any pair of nodes, considering the observed shortest paths^29^, will be used in this study.

Based on the divergence observed in the literature, as well as the lack of studies investigating the training of elite athletes at high altitude, this study aimed to (1) evaluate the blood profile before, during and after the LHTH program; (2) investigate the effects of 30 days of altitude training on aerobic, anaerobic and performance parameters at two different times after returning to low altitude (5–6 and 15–16 days); and (3) evaluate the interconnection of markers beyond conventional statistics with a complex network model analysis. Our hypothesis is that Paralympic athletes will improve aerobic performance after returning to altitude, will have altered the classic hematological parameters from this LHTH intervention and such alterations will be reinforced by the centrality metrics studied from complex networks analysis.

## Materials and methods

### Subjects

Ten elite runners (medium and long distances) of the Brazilian Paralympic athletics team participated in this study. The sample included eight men and two women: four athletes with visual impairments, two athletes with physical disabilities without specific impairment for the motor gesture in a race and four athletes without disability (i.e., guide athlete) (35 ± 8 years, 63.2 ± 5.5 kg, 172.5 ± 4.6 cm). The participation of the athletes in our study had the written consent from the Brazilian Paralympic Committee (CPB), which authorized the use of data from its database. All subjects had high-performance, with specific training experience of at least 10 years, and participating in world-level athletic competitions and Paralympic games. All participants were over 18 years of age and informed consent was obtained from all subjects who signed it to participate in the study. The research was conducted in accordance with the ethical recommendations of the Declaration of Helsinki and all experiments were approved by the Research Ethics Committee of the Faculty of Medical Sciences of the University of Campinas (protocol CAAE number 16576619.2.0000.5404).

### General procedures

**Figure 1:**
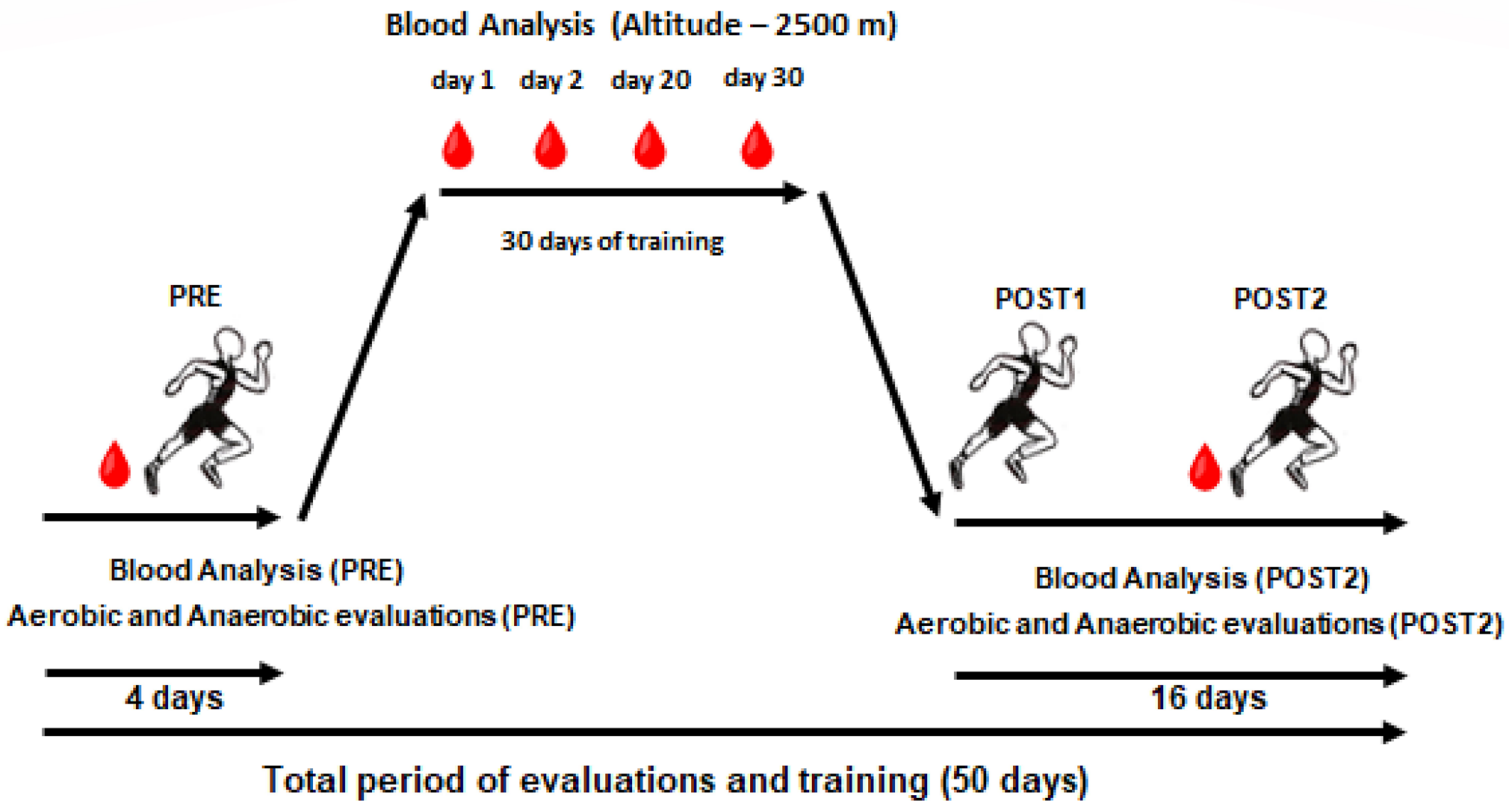
Hematological and physical performance evaluations performed before (PRE), the ascent to altitude, during the stay in Colombia (2500 m) and after returning to lower altitudes (POST). Procedures performed during the entire period of training and evaluations. Assessment schedules: PRE-days -2 and -1 (before the altitude period), POST1-days 5 and 6 (after the altitude period), POST2-days 15 and 16 (after the altitude period). For these moments, the sequential tests performed were anaerobic (AO30) and aerobic (3000 m), respectively.

The physical evaluations (aerobic and anaerobic) were performed before (PRE) and after (POST) altitude training at 2500 m, for 30 days. All physical evaluations were conducted at low altitude, in the São Paulo city (approximately 760 m at sea level). Blood tests were carried out before altitude training (PRE), during altitude training (ALT), and after (POST) (Fig. 1).

Before and after altitude training, the athletes were subjected to aerobic evaluations at the distance of 3000 m and to anaerobic evaluations of maximum power, using the 30 s all-out test in semi-tethered running (AO30). In the PRE phase, the AO30 and 3000 m aerobic tests were conducted 1 and 2 days before starting altitude training (PRE). In the POST phase, the anaerobic test was performed 5 and 15 days after returning from the altitude (POST1 and POST2, respectively), and the 3000 m races took place 6 and 16 days after returning (POST1 and POST2, respectively). As will be further detailed, before, during and after altitude training, athletes were also subjected to blood sample collection for hematological analysis.

### Physical training

The altitude training was carried out in Colombia, at 2500 m. Throughout the period, the athletes and the technical team remained in the same residence, which facilitated controlling nutrition, hydration and post-workout recovery. During the entire training period, athletes were accompanied by a multidisciplinary team composed of physiotherapist, massage therapist, nutritionist and psychologist.

**Figure 2:**
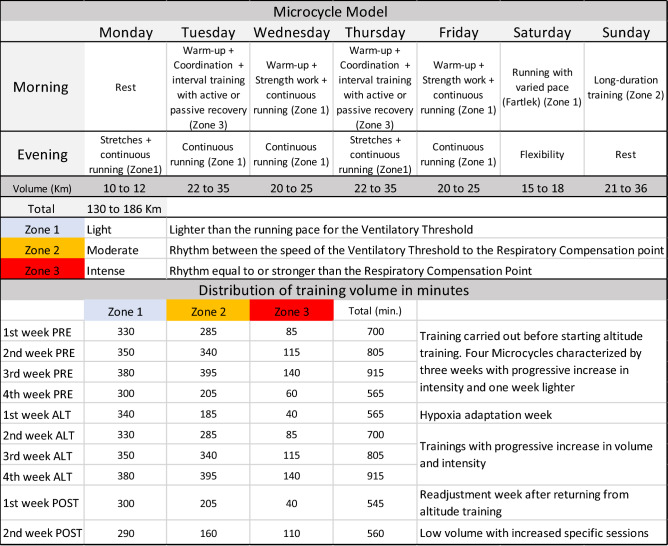
Training model (microcycle) used during altitude training, with details on intensity and quantity in minutes, regarding the training performed 4 weeks before, during, and 2 weeks after altitude training.

The training strategies presented in Fig. 2 included 7-day microcycles, with 12 training sessions: two sessions characterized by interval training (greater intensity), two strength training sessions in the gym, one training session with greater volume, two flexibility sessions and five sessions of lighter training (i.e., regenerative), amounting to 130–186 km (weekly). Regardless of disability or gender, the athletes do not present important limitations in the motor gesture to the running, and because they are athletes with a high-technical level, the training structure was similar for all participants. The training program respected the acclimatization period, with the training sessions taking place with progression of intensity and quantity during the altitude period. The training intensity was determined based on the evaluations of 3000 m, following the observations of Lourenço et al.^30^. According to this author, results coming from a maximum run of 3000 m can provide information to determine different intensity zones; that is, Zone 1, running velocity referring to the ventilatory threshold (vLT); Zone 2, between vVT and respiratory compensation point (vRCP), and Zone 3, above vRCP^30^. Four weeks before starting the training, as well as at the training period at 2500 m, the exercises were characterized by 3-week microcycles, with progressive increase in workload, followed by a less intense week. At altitude, endurance and interval training with distances equal to or greater than 400 m were performed in the same way as is performed at low altitudes, considering the total running volume. However, the intensities were reduced from 10 to 8% in the first week, 6% in the second week and 3% in the other days at 2500 m. On the other hand, speed stimuli with distances shorter than 400 m, as well as muscle strengthening training, did not change when compared to training performed at low altitudes. In the first week after returning, sessions were performed with reduced volume and intensity, respecting the readaptation period. Then, in the second week after returning, the training was characterized by an increase in specific exercises, with a decrease in quantity and an increase in intensity (Fig. 2).

### Running evaluations

#### 3000 m test for performance evaluation

All 3000 m tests were carried out in São Paulo city, on a synthetic rubber track with official measures (400 m). The tests were always carried out in the morning, after a standard warm-up routine of 15 min of continuous running at 12–13 km h^−1^, 5 min of dynamic stretching, 10 min of coordination exercises and three to four repetitions of 80 m with progressive rhythm running, followed by 5 min for the return of physiological responses to rest. The total time of the 3000 m tests was recorded with a Casio digital stopwatch, model Hs-80tw-1DF, used for comparisons between PRE, POST1 and POST2.

#### Anaerobic test: all-out of 30 s (AO30) in semi-tethered running

Of the ten participants who carried out the 3000 m tests and blood tests, eight were also submitted to a 30-s semi-tethered running (AO30). In this case, six men and two women were evaluated (34.3 ± 9.2 years, 63.5 ± 6.1 kg, 170.5 ± 4.2 cm). Among these, three athletes had visual impairment, one athlete, mild physical impairment in the upper limb, and four were guide athletes, that is, able-bodied individuals.

**Figure 3:**
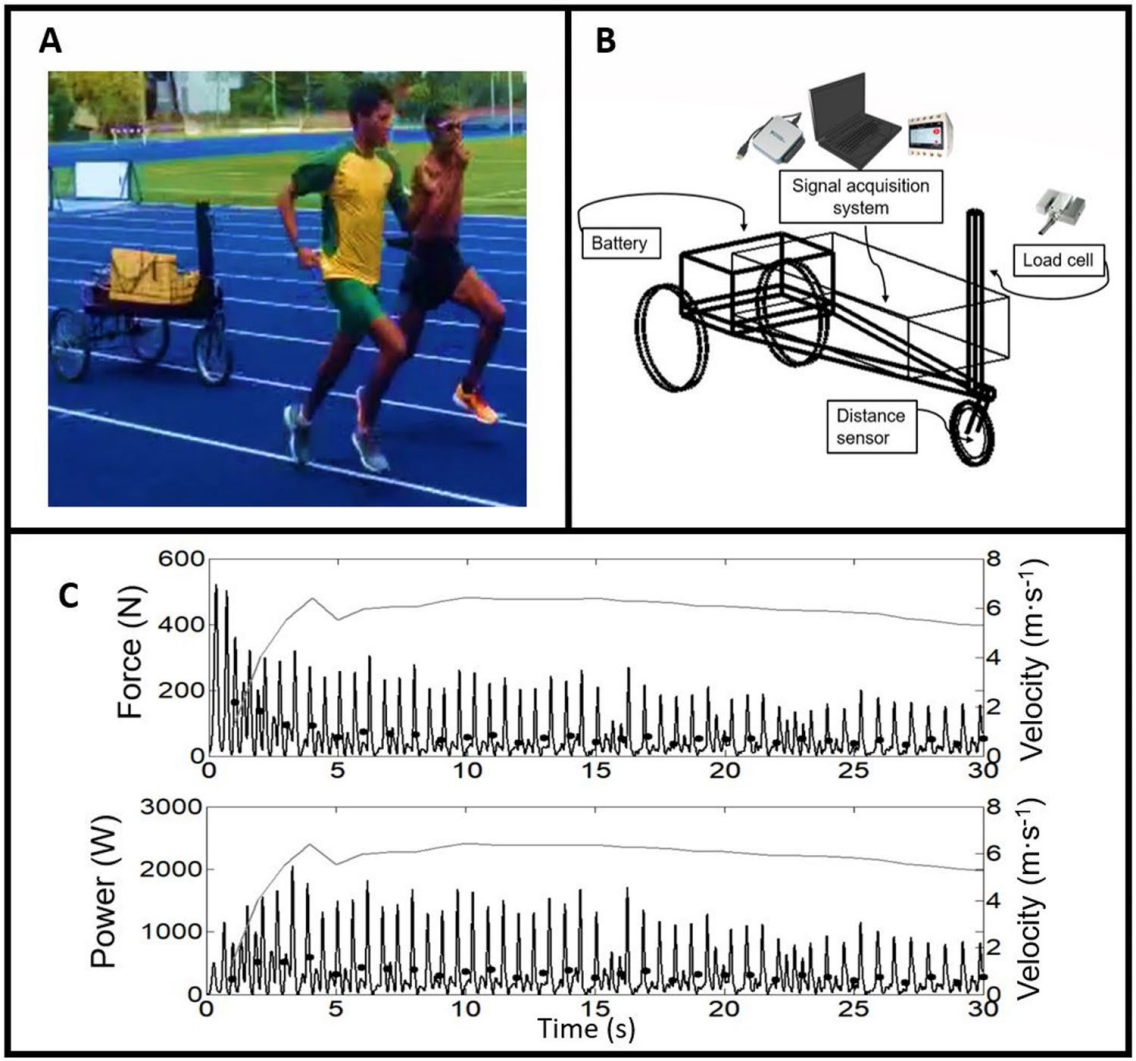
(**A**,**B**) illustrate the apparatus used during the tethered running test. (**C**) Exemplifies the signals of force, power and velocity, recorded during the test (AO30); black lines representing power and force are the raw data, black dots are the averages every second, and gray lines represent the Y axis velocity (on the right). The two athletes represented in the figure signed statements authorizing the publication of their images in an open access scientific journal.

To assess the velocity, force and consequently the mechanical power of the running, the athletes were submitted to the 30-s test in semi-tethered running (AO30). The test was carried out with the athletes tethered to a sensorized equipment (variable-resistance car—VRC), to obtain records with high-frequency signals that were recorded in an on-board computer. The prototype (VRC) was developed in the Laboratory of Applied to Sport Physiology (LAFAE), Unicamp-Limeira-SP (Fig. 3)^31^. In this assessment protocol, athletes were instructed to run as fast as possible for 30 s, attached to the VRC by means of a traction belt and a steel cable, connected to a calibrated dynamometer, allowing the system to measure the application of drag force from records and signals (1000 Hz) using the LabView-SignalExpress software. A disk brake in both rear VRC wheels enabled the imposition of resistance, set at 9% of body mass^31^. The velocity was obtained from the Hall-effect sensor attached to the front fork (which fixed the wheel), and four magnets were inserted equidistantly into the wheel rim (16 in. in diameter), capturing and sending signals to the computer each time it the magnets crossed the sensor. The force and Hall-effect sensors signals were synchronized and analyzed using a dedicated algorithm in the MatLab environment. Thus, the power was obtained by the product of the force and velocity data of the VRC ergometer. All AO30 tests were performed on the official athletics track, at low altitude (760 m), 2 days before the start of altitude training (PRE), as well as 5 days and 15 days after returning to lower altitudes (POST1 and POST2, respectively). During the AO30, the participants were verbally encouraged to maintain the maximal velocity throughout the whole all-out test. The maximal, mean and minimum of power, force and velocity were used as measurements, as well as fatigue indexes (FI) for each one of these mechanical parameters. The force and power parameters were normalized by athlete body mass. The FI was obtained as the decay (%) from the highest to the lowest value.

#### Blood analyses

For the blood samples collection, the athletes underwent arterial puncture to take 10 mL of blood from the antecubital artery, in order to determine Hemoglobin concentrations (Hb—g dL^−1^), Hematocrits (Hct—%), Red Blood Cells (RBC—M mm^−3^), Mean Corpuscular Volume (MCV—fL), Mean Corpuscular Hemoglobin (MCH—pg), Mean Corpuscular Hemoglobin Concentration (MCHC—g dL^−1^), Red Cell Distribution Width (RDW—%), Leukocytes (White Blood Cells—WBC—10^3^ mm^−3^), Lymphocytes (%), Monocytes (%), Neutrophils (%) and Platelets (10^6^ mm^−3^). These analyses were performed at the following phases: PRE (3 days before starting altitude training); during the LHTH program, on the first day (d1), on the 12th day (d12), on the 20th day at altitude (d20), on the 30th day at altitude (d30) and 15 days after returning (POST2). The collections were carried out by professionals in a laboratory, under consistent conditions, after an 8-h fasting.

### Statistical procedures

Results are presented as means ± standard deviation (SD). Data normality and homogeneity were initially confirmed by Shapiro–Wilk and Levene tests, respectively. To compare the results at different time periods, we used one-way ANOVA for repeated measures and the Newman–Keuls post-hoc test (p ≤ 0.05). We used Statistica 7.0 (Statsoft Inc.) and Excel (Microsoft Office, 2019) software for statistical analyses. Results were displayed as box plots using GraphPad Prism software. The effect size (Cohen's d) for pairwise comparisons was determined from the difference between means divided by pooled SD. Additionally, eta squared (η^2^) for each ANOVA was determined dividing the sum of squares for each effect (SS_effect_) by the total sum of squares (SS_total_). Product–moment correlation analyzes were used for all parameters normalized by the body mass, and the investigation with the complex networks model identified measures of network centrality for two scenarios: PRE and POST2. This analysis was performed with Gephi (version 0.9.2, https://gephi.org/), implemented in JAVA (version 8) programming language applying the Fruchterman-Reingold layout^32^, after being processed step-by-step in algorithms specifically built for the study in MatLab environment (MathWorks Inc.). The complex networks were built considering non-directed relations and with weight, graphs G = (V, E, w), for both scenarios, where V (vertices) represents nodes and E (edges), the interactions between two variables in this network. Weights were calculated by the correlation value^33^, according to Pereira et al.^23^, using critical r of 0.5 (moderate correlation). The centrality metrics adopted for the analysis were the *pondered degree*, *pagerank* and *betweenness*.

The comparisons between two complex networks, built considering the PRE and POST2 scenarios and hereinafter referred to as G_PRE_ and G_POST2_, were performed using the weighted Jaccard distance^34,35^, in two fashions toward having a graph- and node-based dissimilarity measurement. In both cases, this measure assumes higher values if two networks or pairs of nodes being compared are dissimilar and lower values if they are similar.

To compute the dissimilarity measure between two complex networks in a graph-based fashion, we computed the weighted Jaccard distance between two networks G_PRE_ and G_POST2_, as suggested by Tantardini et al.^35^. More precisely, let G_PRE_ = (V_PRE_, E_PRE_) and G_POST2_ = (V_POST2_, E_POST2_) two networks aligned and with adjacency matrices adj(G_PRE_) = A and adj(G_POST2_) = B, respectively. The weighted Jaccard distance is d_GWJ_ = 1 − J_GW_(G_PRE_, G_POST2_), where J_GW_ is the Jaccard index defined as:

$$J_{GW} (G_{PRE} ,G_{POST2} ) = \left\{ {\begin{array}{*{20}l} {\frac{{\sum\nolimits_{(i,j) \in V} {\min (A_{i,j} ,B_{i,j} )} }}{{\sum\nolimits_{(i,j) \in V} {\max (A_{i,j} ,B_{i,j} )} }},} & {{\text{if}}\;\sum\nolimits_{(i,j) \in V} {\max (A_{i,j} ,B_{i,j} )} > 0} \\ {1,} & {{\text{otherwise}}} \\ \end{array} } \right\}$$

where V = V_PRE_ = V_POST2_. In turn, the node-based measurement for a node of interest v was achieved by computing the weighted Jaccard similarity as suggested by Fender et al.^34^:

$$J_{NW} (\nu ) = \sum\limits_{z \in N(v)} {\frac{\Gamma (N(v) \cap N(z))}{{\Gamma (N(v) \cup N(z))}}} ,\quad \Gamma (U) = \sum\limits_{x \in U} {w(x)}$$

where w(x) refers to a centrality measurement of the node x. Thus, the node-based weighted Jaccard distance can be defined as d_NWJ_ = 1 − J_NW_(v), which give us a measurement of how a node of interest v is dissimilar to their neighborhood. In this study, we used these metrics to measure how the POST2 phase affected some variables considered in our study.

## Results

### 3000 m tests

**Figure 4:**
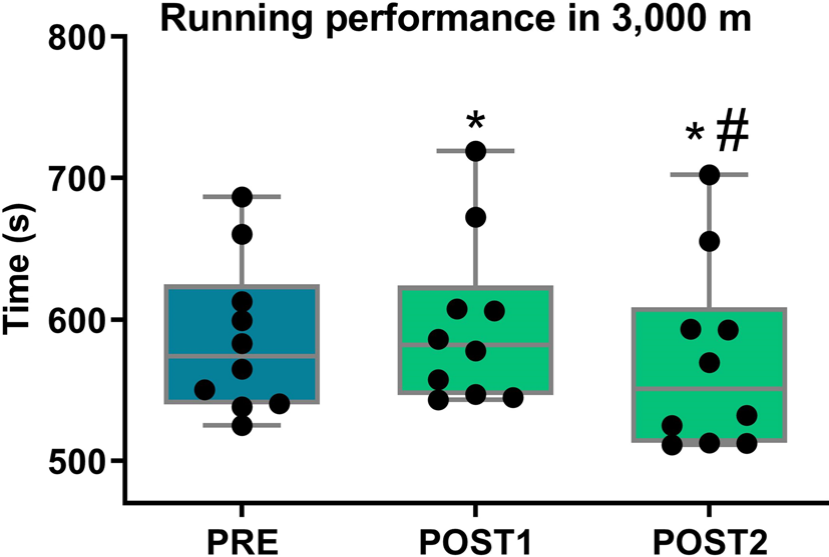
Running performance in 3000 m before (blue bar) and at two moments after altitude exposure (green bar). Box plots depict median value (line in the box) with box edges representing 25th–75th percentiles and whiskers showing range of values (min to max). *Difference of PRE, ^#^difference of POST1 (according to repeated‐measures ANOVA using the Newman–Keuls post-hoc test).

The performance results in the 3000 m running test (Fig. 4), showed that nine of the ten participants presented their best result at POST2 (i.e., at 16 days after returning from altitude training). Another point observed was that, when compared to PRE, nine athletes decreased their performance on the 6th after returning (POST1).

The results of the times for the PRE, POST1 and POST2 groups were respectively 586.1 ± 54.0, 596.2 ± 58.5, and 570.7 ± 66.0 s, and the ANOVA analysis revealed that these three periods differed significantly (F = 15.4, p < 0.01, η^2^ = 0.03). Post-hoc comparisons indicated a significant reduction of performance (higher time in 3000 m running) at POST1 as compared to PRE (p < 0.05, Cohen’s d = 0.18). A shorter time in the 3000 m running (performance improvement) was observed at POST2 when compared to POST1 (p < 0.001, Cohen’s d = 0.41) and PRE (p < 0.01, Cohen’s d = 0.26). The correlation analyses for PRE, POST1 and POST2 were significant in all cases: PRE and POST1 (r = 0.96 p < 0.001), PRE and POST2 (r = 0.98, p < 0.01), POST1 and POST2 (r = 0.98, p < 0.01), suggesting consistent performances in the evaluations.

### Running power in AO30

**Figure 5:**
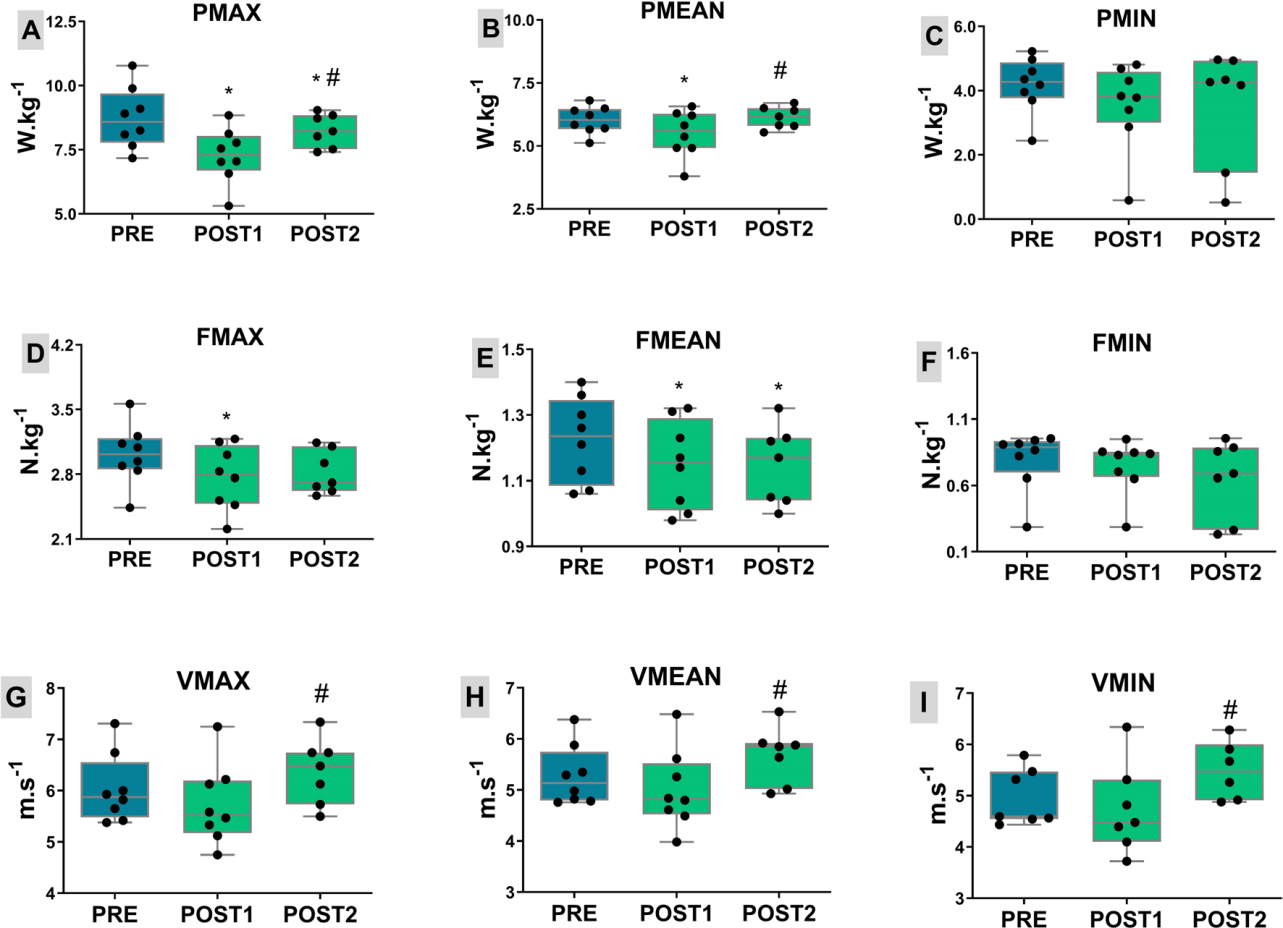
Mechanical results obtained during tethered running before (PRE—blue bar) and at two moments after altitude exposure (POST—green bar). Box plots depict median value (line in the box) with box edges representing 25th–75th percentiles and whiskers showing range of values (min to max) for maximum power (PMAX, **A**), mean power (PMEAN, **B**), minimum power (PMIN, **C**), maximum force (FMAX, **D**), mean force (FMEAN, **E**), minimum force (FMIN, **F**), maximum velocity (VMAX, **G**), mean velocity (VMEAN, **H**) and minimum velocity (VMIN, **I**). Power and force data were normalized by body mass. *Different of PRE, ^**#**^different of POST1 (according to repeated‐measures ANOVA using Newman–Keuls post-hoc test).

Regarding the anaerobic tests with AO30, the means of maximum relative power were 8.73 ± 1.19 (PRE), 7.28 ± 1.07 (POST1) and 8.25 ± 0.64 W kg^−1^ (POST2). ANOVA analysis showed that these three periods differed significantly (F = 20.15, p < 0.01, η^2^ = 0.30). Post-hoc comparisons indicated significant difference between PRE and POST1 (p < 0.01, Cohen’s d = 1.28), PRE and POST2 (p < 0.05, Cohen’s d = 0.52), and between POST1 and POST2 (p < 0.01, Cohen’s d = 1.14) (Fig. 5A). The results for mean relative power (W kg^−1^) are presented in panel B of Fig. 5; with ANOVA showing significant differences among periods (F = 8.46, p < 0.01, η^2^ = 0.16). The mean values were 6.03 ± 0.55 (PRE), 5.49 ± 0.92 (POST1) and 6.13 ± 0.43 W kg^−1^ (POST2). Significant differences were observed between PRE and POST1 (p < 0.01, Cohen’s d = 0.74), and between POST1 and POST2 (p < 0.01, Cohen’s d = 0.95). Post-hoc comparisons indicated no significant difference in mean relative power between PRE and POST2 (p = 0.79, Cohen’s d = 0.21). With regard to minimum relative power (Fig. 5C) ANOVA did not show difference among the three time periods (F = 2.61, p > 0.05, η^2^ = 0.05). The mean values were 4.18 ± 0.86 (PRE), 3.53 ± 1.30 (POST1) and 3.52 ± 1.71 W kg^−1^ (POST2), with pairwise comparisons showed no significant differences between PRE and POST1 (p = 0.11, Cohen’s d = 0.58), between PRE and POST2 (p = 0.11, Cohen’s d = 0.50) and between POST1 and POST2 (p = 0.67, Cohen’s d = 0.01). Regarding power fatigue index (%), no differences were observed among the three periods (F = 0.15, p > 0.05, η^2^ = 0.02). The mean values of power fatigue index were 50.25 ± 8.04 (PRE), 48.20 ± 9.42 (POST1) and 52.43 ± 13.92 (POST2), with no significant difference between PRE and POST1 (p = 0.75, Cohen’s d = 0.23), between PRE and POST2 (p = 0.81, Cohen’s d = 0.20) and between POST1 and POST2 (p = 0.84, Cohen’s d = 0.36).

Regarding body mass, the mean values were 63.20 ± 5.61 kg (PRE), 62.97 ± 5.74 kg, (POST1) and 63.16 ± 5.63 kg (POST2). When compared to PRE, athletes decreased their body mass at POST1 (p = 0.01, Cohen’s d = 0.04). There is no difference in body mass between PRE and POST2 (p = 0.57, Cohen’s d = 0.01), but post-hoc comparisons indicated significant difference in body mass at POST1 as compared to POST2 (p = 0.01, Cohen’s d = 0.03).

### Running force in AO30

Figure 5 (panel D), shows the relative results of maximum force (N kg^−1^), obtained in AO30. The mean values obtained were 3.01 ± 0.32 (PRE), 2.77 ± 0.34 (POST1) and 2.82 ± 0.23 (POST2), with ANOVA showing significant differences among time periods (F = 4.17, p < 0.05, η^2^ = 0.12). Post hoc comparisons show significant differences between PRE and POST1 (p < 0.05, Cohen’s d = 0.73). We found no significant differences between PRE and POST2 (p > 0.05, Cohen’s d = 0.70) and between POST1 and POST2 (p = 0.54, Cohen’s d = 0.17).

With regard to mean relative force (Fig. 5E), ANOVA analysis showed that these three periods differed significantly (F = 16.16, p < 0.01, η^2^ = 0.08). The mean relative force (N kg^−1^) were 1.22 ± 0.13 (PRE), 1.15 ± 0.13 (POST1) and 1.15 ± 0.12 (POST2), being significantly different between PRE and POST1 (p < 0.01, Cohen’s d = 0.57), and between PRE and POST2 (p < 0.01, Cohen’s d = 0.61). We found no significant differences in mean relative force between POST1 and POST2 (p = 0.21, Cohen’s d = 0.01).

ANOVA did not show a significant difference among the three periods on minimum relative force (F = 2.03, p > 0.05, η^2^ = 0.06). The main values of minimum relative force (Fig. 5F) were 0.79 ± 0.21 (PRE), 0.74 ± 0.22 (POST1) and 0.65 ± 0.34 (POST2), with pairwise comparisons showed no significant differences between PRE and POST1 (p = 0.37, Cohen’s d = 0.22), between PRE and POST2 (p = 0.15, Cohen’s d = 0.56) and between POST1 and POST2 (p = 0.29, Cohen’s d = 0.39). The force fatigue index (%) values were 72.20 ± 5.22 (PRE), 71.86 ± 5.04 (POST1) and 75.35 ± 7.67 (POST2), and ANOVA did not show a significant difference among the three periods (F = 0.52, p > 0.05, η^2^ = 0.02). Post hoc comparisons showed no significant difference between PRE and POST1 (p = 0.95, Cohen’s d = 0.07), between PRE and POST2 (p = 0.41, Cohen’s d = 0.49) and between POST1 and POST2 (p = 0.64, Cohen’s d = 0.55).

### Running velocity in AO30

The maximum velocity observed in AO30 are presented in Fig. 5, panel G. The results were 6.03 ± 0.67 (PRE), 5.73 ± 0.78 (POST1) and 6.38 ± 0.64 m s^−1^ (POST2), and ANOVA analysis showed that these three periods differed significantly (F = 4.48, p < 0.05, η^2^ = 0.13). Post hoc comparisons show significant differences between POST1 and POST2 (p < 0.05, Cohen’s d = 0.91). We found no significant differences between PRE and POST1 (p = 0.11, Cohen’s d = 0.42) and between PRE and POST2 (p = 0.22, Cohen’s d = 0.53).

Regarding mean velocity values, we found 5.28 ± 0.58 (PRE), 5.01 ± 0.78 (POST1) and 5.68 ± 0.56 m s^−1^ (POST2), with ANOVA showing significant differences among periods (F = 5.17, p < 0.05, η^2^ = 0.16). Post hoc comparisons show significant differences between POST1 and POST2 (p = 0.01, Cohen’s d = 1.02). We found no significant differences between PRE and POST1 (p = 0.13, Cohen’s d = 0.40) and between PRE and POST2 (p = 0.13, Cohen’s d = 0.71) (Fig. 5H).

The minimum velocity was 4.96 ± 0.56 (PRE), 4.74 ± 0.86 (POST1), and 5.49 ± 0.56 m s^−1^ (POST2), with ANOVA showing significant differences among periods (F = 5.54, p = 0.02, η^2^ = 0.19). We found significant differences in minimum velocity between POST1 and POST2 (p = 0.01, Cohen’s d = 1.05) (Fig. 5I). Still regarding minimum velocity, no significant differences were noted between PRE and POST1 (p = 0.21, Cohen’s d = 0.31) and between PRE and POST2 (p = 0.07, Cohen’s d = 0.95). The velocity fatigue index (%) values were 16.44 ± 4.92 (PRE), 18.02 ± 6.49 (POST1) and 13.55 ± 5.02 (POST2), and ANOVA did not show a significant difference among the three periods (F = 3.09, p > 0.05, η^2^ = 0.11). Post hoc comparisons showed no significant difference between PRE and POST1 (p = 0.26, Cohen’s d = 0.28), between PRE and POST2 (p = 0.21, Cohen’s d = 0.58) and between POST1 and POST2 (p = 0.06, Cohen’s d = 0.78).

### Blood test results

**Figure 6:**
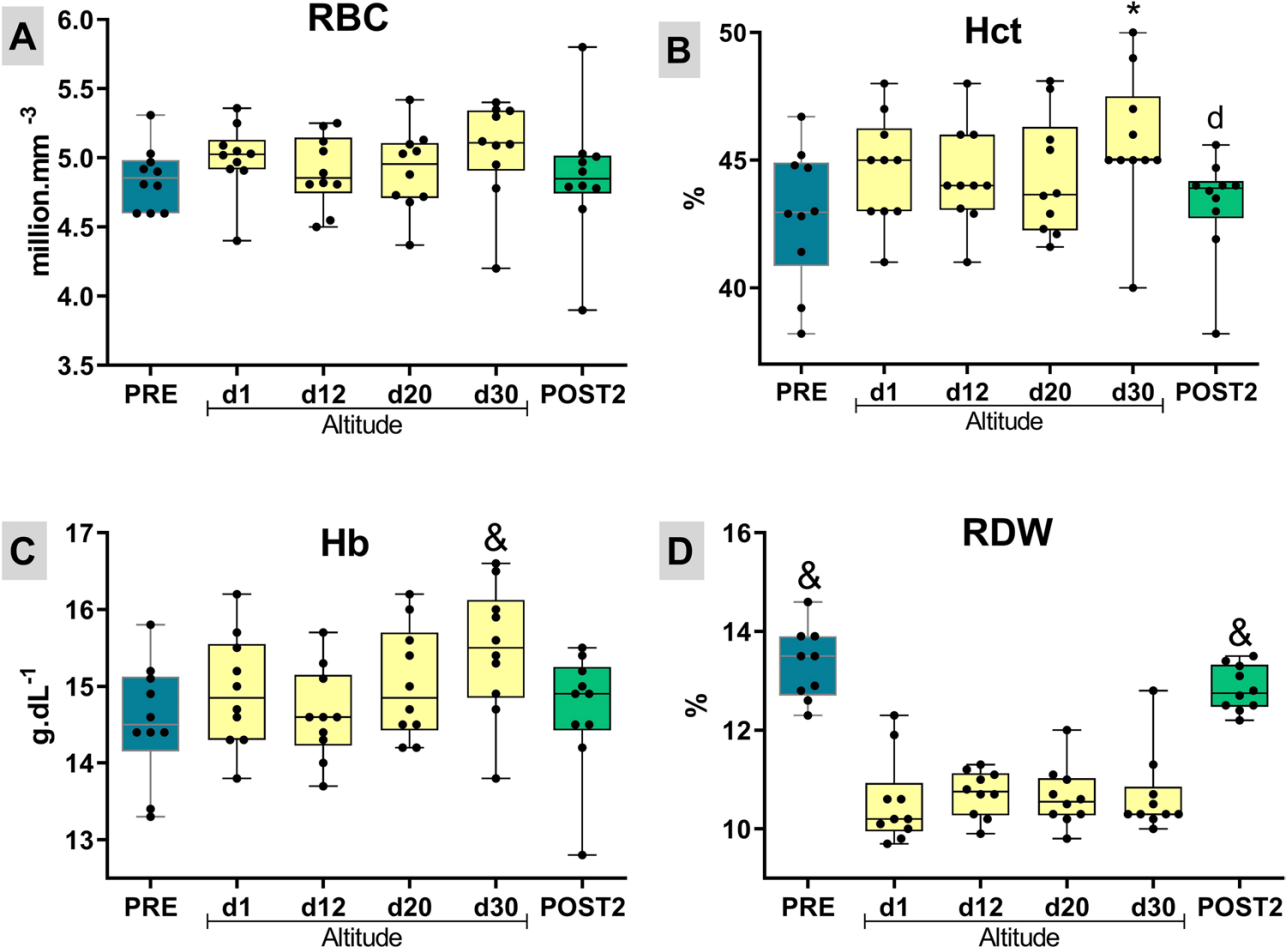
Red blood cells—RBC (**A**), hematocrit—Hct (**B**), Hemoglobin—Hb (**C**). (**D**) The red blood cell distribution width (RDW). Box plots depict median value (line in the box) with box edges representing 25th–75th percentiles and whiskers showing range of values (min to max). *Different of PRE; ^d^different of d30; ^&^different of all other groups (according to repeated‐measures ANOVA, using the Newman–Keuls post-hoc test).

Regarding to RBC, ANOVA did not show a significant difference among periods (F = 2.22, p > 0.05, η^2^ = 0.05). The results for RBC (M mm^−3^) were 4.9 ± 0.2 (PRE), 5.0 ± 0.3 (d1), 4.9 ± 0.3 (d12), 4.9 ± 0.3 (d20), 5.1 ± 0.4 (d30) and 4.9 ± 0.5 (POST2), with no significant differences found (Fig. 6A). The hematocrit results, in percentage (%), are presented in Fig. 6, panel B. The obtained values were 42.9 ± 2.6 (PRE), 44.6 ± 2.1 (d1), 44.3 ± 1.9 (d12), 44.3 ± 2.3 (d20), 45.7 ± 2.7 (d30) and 43.3 ± 2.0 (POST2), significant differences among periods (F = 4.48, p < 0.05, η^2^ = 0.14). Significant differences were observed between the PRE and d30 (p < 0.001, Cohen’s d = 1.04), and between D30 and POST2 (p < 0.001, Cohen’s d = 1.03).

Regarding hemoglobin changes (g dL^−1^), Fig. 6C shows that all evaluated moments were statistically lower (p < 0.05) than those found at the end of the 30th day (d30) at altitude. ANOVA analysis showed significant differences among moments (F = 6.21, p < 0.01, η^2^ = 0.16). The results for the period were 14.55 ± 0.8 (PRE), 14.93 ± 0.7 (d1), 14.63 ± 0.6 (d12), 15.03 ± 0.7 (d20), 15.47 ± 0.9 (d30) and 14.69 ± 0.8 (POST2). When comparing PRE with D30, the results showed a 6.32% increase in hemoglobin (p < 0.01, Cohen’s d = 1.13). However, in POST2, 15 days after altitude training, the values of hematological parameters returned to the PRE values.

The results for RDW are presented in percentage (%) in Fig. 6D: 13.33 ± 0.7 (PRE), 10.54 ± 0.9 (d1), 10.72 ± 0.5 (d12), 10.65 ± 0.6 (d20), 10.67 ± 0.8 (d30) and 12.84 ± 0.5 (POST2), with ANOVA showing significant differences among moments (F = 84.07, p < 0.01, η^2^ = 0.75). Significant differences were observed between the PRE moments and d1 moments (p < 0.001, Cohen’s d = 3.45), d12 (p < 0.001, Cohen’s d = 4.35), d20 (p < 0.001, Cohen’s d = 3.98), d30 (p < 0.001, Cohen’s d = 3.40), POST2 (p = 0.03, Cohen’s d = 0.82). We found a difference between POST2 and d1 (p < 0.001, Cohen’s d = 3.45), d12 (p < 0.001, Cohen’s d = 4.61), d20 (p < 0.001, Cohen’s d = 4.10), d30 (p < 0.001, Cohen’s d = 3.37). Other results of hematological variables such as MCV, MCH, MCHC, leukocytes, lymphocytes, monocytes, neutrophils, and platelets are shown in Table 1.

**Table 1:** Mean corpuscular volume (MCV), mean corpuscular hemoglobin (MCH), mean corpuscular hemoglobin concentration (MCHC), leukocytes, lymphocytes, monocytes, neutrophils and platelets from the before moment (PRE), at four moments during altitude exposure and after return to low altitude (POST2).

	PRE	d1	d12	d20	d30	POST2	ANOVA
MCV (fL)	89.5 ± 4.2	88.9 ± 2.6	90.1 ± 3.3	89.8 ± 3.4	89.9 ± 3.7	90.2 ± 3.8	F = 1.13, p = 0.35, η^2^ = 0.01
MCH (pg)	30.5 ± 1.5	29.4 ± 1.0	29.6 ± 1.5	30.3 ± 1.4	29.9 ± 1.4	30.3 ± 1.4	F = 2.44, p = 0.04, η^2^ = 0.08
MCHC (g dL^−1^)	34.0 ± 0.7^abd^	33.3 ± 0.5	33.0 ± 0.4	33.8 ± 0.3^ab^	33.5 ± 0.4	33.6 ± 0.4^b^	F = 7.37, p < 0.01, η^2^ = 0.37
Leukocytes (10^3^ mm^−3^)	5945.0 ± 1587.0	5870.0 ± 1123.0	6050.0 ± 1422.2	6030.0 ± 969.6	6410.0 ± 1350.3	6665.0 ± 1351.5	F = 1.18, p = 0.33, η^2^ = 0.04
Lymphocytes (%)	33.7 ± 6.2^abcd^	51.6 ± 9.9	48.6 ± 13.5	47.3 ± 6.3	46.5 ± 6.9	33.5 ± 7.9^abcd^	F = 13.40, p < 0.01, η^2^ = 0.42
Monocytes (%)	8.8 ± 3.3^abcd^	3.9 ± 1.4	4.5 ± 1.3	2.6 ± 0.5	2.6 ± 0.5	5.8 ± 1.8*^cd^	F = 16.95, p < 0.01, η^2^ = 0.62
Neutrophils (%)	48.8 ± 12.7	44.3 ± 10.6	45.8 ± 13.1	47.3 ± 6.5	48.7 ± 8.6	57.8 ± 9.4*^abcd^	F = 4.42, p < 0.05, η^2^ = 0.16
Platelets (10^6^ mm^−3^)	241.6 ± 46.4^abcd^	295.2 ± 58.6	316.8 ± 50.2	328.4 ± 46.6	316.5 ± 45.6	266.5 ± 36.6^abcd^	F = 12.11, p < 0.01, η^2^ = 0.31

Data are in mean ± standard deviation. *Different of PRE; ^a^different of d1; ^b^different of d12; ^c^different of d20; ^d^different of d30 (according to repeated‐measures ANOVA, using Newman–Keuls post hoc test).

### Analysis by complex networks

**Figure 7:**
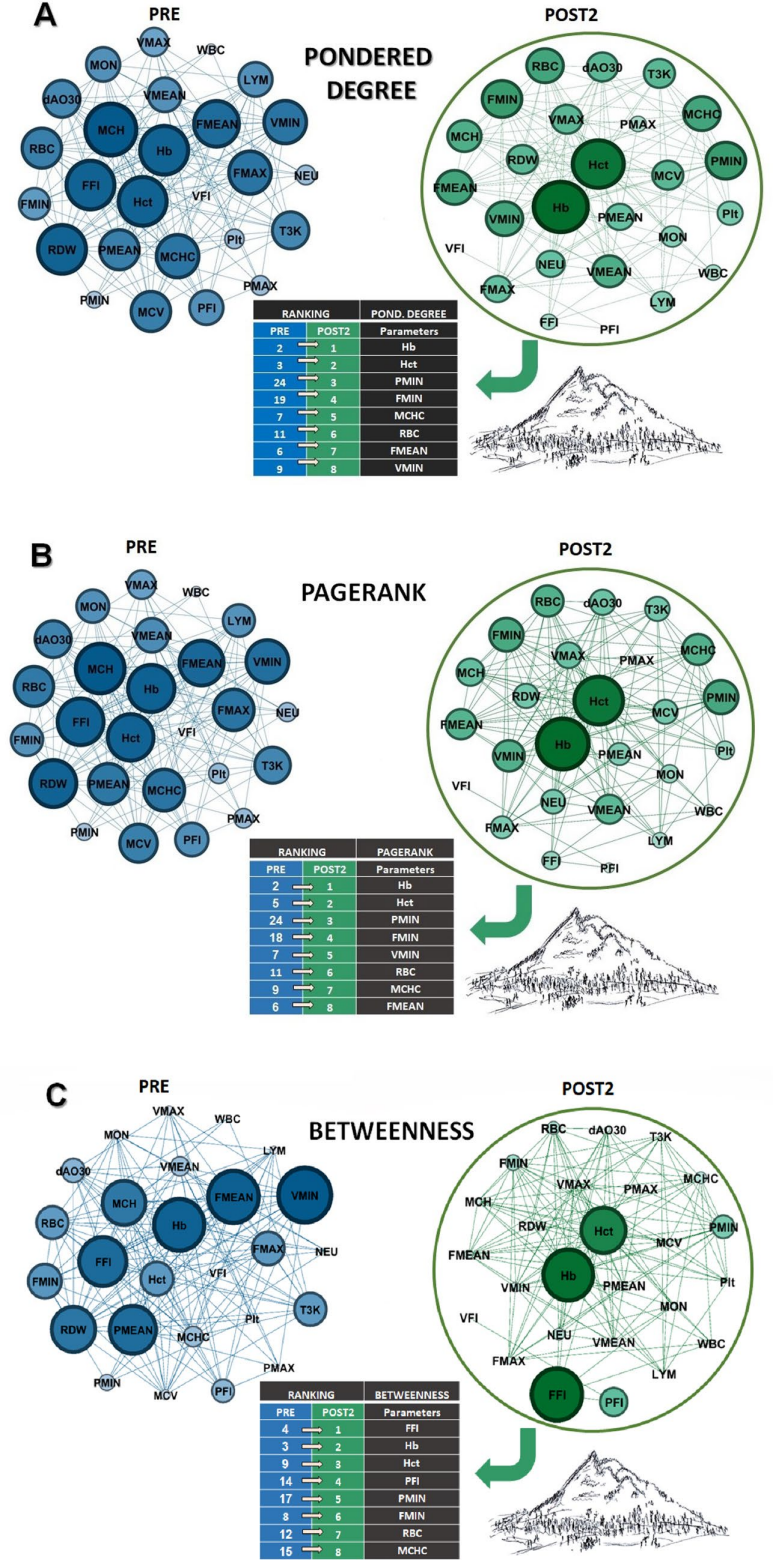
Centrality metrics in complex networks model. Panels (**A**–**C**) represent, respectively, the *pondered degree*, *pagerank* and *betweenness* graphs. The central table presents the positions of centrality for each metric in the two scenarios involved, being PRE immediately before the ascent to the altitude (2500 m) and POST2, 15 days after returning from altitudes.

Figure 7 presents graphs for the centrality metrics *pondered degree* (panel A), *pagerank* (panel B) and *betweenness* (panel C). At the center, among the graphs referring to the two scenarios studied (PRE and POST2), are presented the positions of the variables in order of importance, within the ranking obtained for each metrics in both scenarios, with the arrows indicating the evolution after the intervention. In total, 26 variables were analyzed in both scenarios, with the first eight positions listed and presented here.

**Figure 8:**
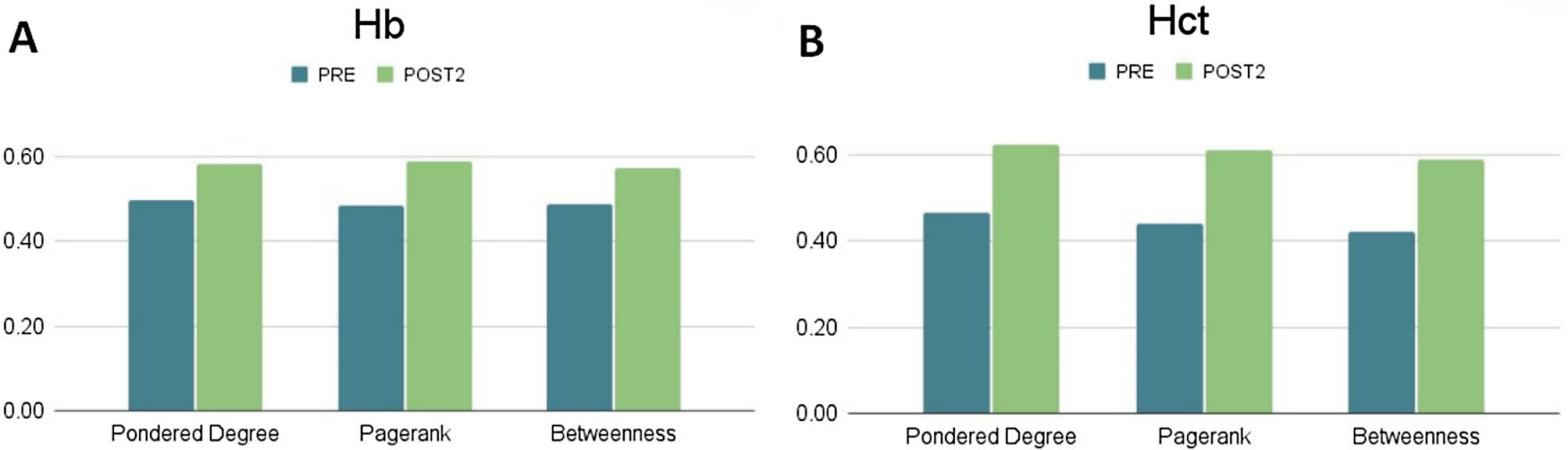
Node-based weighted Jaccard distances. (**A**) and (**B**) show the weighted Jaccard distances computed for the Hb and Hct variables, respectively, weighted by the *pondered degree*, *pagerank* and *betweenness* centrality measures.

The comparison between networks built from PRE and POST2 scenarios showed a dissimilarity of d_GWJ_ = 0.852. Furthermore, Fig. 8 shows a comparison between graphs built from the PRE and POST2 scenarios using the node-based weighted Jaccard distance for the Hb and Hct variables. We also observed that both variables presented a higher dissimilarity among their neighbors for the POST2 scenario, in comparison to the PRE scenario.

## Discussion

The main findings of our study show a positive effect of LHTH on the hematological responses of athletes during altitude training and increase in anaerobic parameters and performance when running after 15–16 days of readaptation to low altitude, suggesting that this strategy is significant for high-performance athletes, in the case of elite runners of the Brazilian Paralympic team. It is important to highlight the quality of the athletes submitted to LHTH for 30 days, as well as the refined training set of physical performance tests used in our study.

The benefits of living high-training high (LHTH) to improve performance at low altitude are controversial. This is due to factors that may directly influence the results: (1) insufficient hypoxic effect to stimulate the total volume of red blood cells/hemoglobin mass, due to insufficient altitude (< 2000 to 2200 m) and/or an insufficient period of exposure, < 3–4 weeks; (2) insufficient training stimuli to improve the function of the neuromuscular and cardiovascular system;( 3) overtraining, stress symptoms and infections^10^. However, when we consider the total time of the different moments of the 3000 m tests, we can state that the effects of altitude and the training performed generated similar adaptations for all the participants, confirming the hypothesis of positive influences of altitude training on aerobic capacity.

In the present study, the altitude of 2500 m was chosen to LHTH for two main reasons. The first is related to the fact that it has been added and suggested that altitudes between 2000 and 2500 m seem "ideal" for erythropoietic, physiological adaptations and endurance performance improvements^2,36^. Chapman et al.^22^, using the LHTL model, when they submitted university runners to four different altitudes (1780, 2085, 2454, and 2800 m) for 4 weeks, they found that only runners living at moderate altitudes (2085 and 2454) increased (2–3%) a 3000 m time trial immediately and 2 weeks after the return to sea level. The second refers to the attempt to guarantee hematological adaptations, but concomitantly, without a great reduction in the absolute intensity of the aerobic training, mainly because the investigated athletes compete in endurance events and the training phase was part of a Paralympic cycle. Studies have clearly shown that during training performed at altitude as the absolute training intensities at the anaerobic threshold and at VO_2peak_ are reduced^37–39^ and this aspect was predicted in our schedule (Fig. 2).

Studies that analyze performance after returning from altitude are scarse^22^. Most evaluate performance before the onset of training and shortly after returning, thus losing important information within a 2–3-week window. In the training model presented in this research, the athletes performed tests of 3000 m before starting training at 2500 m (altitude) and repeated the tests with 6 and 16 days after altitude exposure. In the tests performed 6 days after returning, nine of the ten athletes showed a decrease in performance. However, 16 days after readaptation at low altitudes, nine of the ten athletes had their best result of the entire season, with a reduction of 15.4 s in the 3000 m race between the initial evaluation and POST2. Similar results were found by Levine and Stray-Gundersen^40^, who reported improvements of 13.4 s in a 5000 m race, 14 and 21 days after returning from altitude—in the case of athletes who performed the LHTL training model. The results in both the LHTH and LHTL models suggest that the effects of altitude training can be maintained for up to 3 weeks.

Levine and Stray-Gundersen^1^ reported that due to lower oxygen availability, stress stimuli tend to occur at reduced intensities, leading to losses in velocity gain. In our study, the results from 30 s semi-tethered running showed that, 5 days after returning from a 30-day phase of training at altitude at 2500 m, six of the eight evaluated athletes had a decrease in velocity and mechanical power, which may be associated with a decrease in intensity during the interval training in hypoxia. On the other hand, after a 15-day rehabilitation phase, seven of the eight evaluated athletes achieved the best outcomes of their careers.

Regarding the force component obtained in AO30, we verified reduced values (p < 0.05) in POST1 and POST2. Of the eight athletes studied, only one did not show lower results in force after the intervals when returning from hypoxia training. A study conducted with humans^41^ aiming to isolate the effect of reduced partial oxygen pressure, with progressive elevation to extreme altitudes (equivalent to the peak of Mount Everest), was conducted in order to verify the behavior of lower and upper limbs muscles at the end of 40 days in simulated hypoxia. The data revealed, by computed tomography, that muscle areas were smaller after chronic exposure to altitude, and that the cross sections of Type I and II fibers were also reduced^41^. Similar results were observed in animals under similar conditions, when the authors observed elevated protein expression of myostatin, with significant muscle atrophy^42^. Vargas-Pinilla^19^ reports that training under hypoxia reduces muscle strength and power, corroborating thus our results. This suggests the need for adjustments in strength training during LHTH periods, since in POST2 all athletes had their best personal result in the 3000 m test. This led to questioning on whether better performance could be observed if muscle strength is not reduced after chronic hypoxia.

To evaluate hematological changes, the athletes in our study were submitted to blood tests before the training (PRE), during exposure to chronic hypoxia at an altitude of 2500 m (days 1, 12, 20 and 30) and 15 days after returning (POST2). In 2016, Garvican-Lewis et al.^6^ questioned whether responses from a longer stay at lower altitudes could cause the same response in hemoglobin concentration, when compared with a shorter exposure time at higher altitudes. More recent studies, using the same statistical approach proposed in a meta-analysis by Gore et al.^43^, suggested a model to determine the hypoxic dose, called "kilometer-hour”, which is the multiplication of km, indicating the altitude used for exposure and the total duration of exposure in hours (km h). The model suggests a 3.3% increase in hemoglobin mass per 1.0 km h. In this sense, our study used 2.5 km of altitude and 720 h, totaling 1.8 km h. Thus, an increase of 6.8% in hemoglobin levels is quite compatible with this prediction. This parameter has been considered an indicator of stress external to hypoxia^6^. In our study, athletes confirmed such adaptations at day 30. Considering our model, with 30 days of LHTH training, amounting to 720 h of exposure at 2500 m, our data showed a gain of 6.8% in hemoglobin levels (g dL^−1^).

Although the immune system is impaired by exposure to hypoxia^44^, Pyne et al.^45^ when investigating the immune responses after 21 days of training at 2102 m, found, in addition to other changes, significant reductions of 38% without the number of leukocytes from elite swimmers, followed by increases in the production of interleukin-1beta induced by mitogen (IL-1beta), IL-4 and interferon-gamma (IFN-gamma). When comparing the smallest changes observed in the members of the technical committee, who were exposed to the same altitude dose, but did not perform the training program, and therefore were used as a control group, these authors concluded that although some immunological parameters are changed with training at moderate altitude, as training-induced changes may be secondary to those induced solely by altitude.

In this study, lymphocytes and platelets increased by 53 and 22% respectively after the first day of altitude exposure (d1) and remained high, but stable, until the end of exposure (d30), with values returning to baseline levels (PRE), after 16 days (POST2) of return to low altitude. In contrast, monocytes reduced by 56% in d1, remained stable until d30 and increased again to values close to PRE, after POST2. Additionally, despite the significant changes in some immunological markers during the LHTH model, the values will remain within the reference ranges. Furthermore, unlike the findings by Pyne et al.^45^, in the present investigation, the leukocytes were not altered during the 4 weeks of LHTH. Brugniaux et al.^44^, when investigating the effects of LHTL performed at different altitudes [1200 (control group), 2500, 3000 and 3500 m], only found a significant increase in the amount of leukocytes when the altitude was 3500 m. Thus, our findings reinforce the results of previous studies that demonstrated that athletes, in general, can tolerate 4 weeks of LHTH, without major changes in the immune system.

Although the effects were satisfactory, we realized that by the time the athletes presented their best results in the 3000 m tests, that is, 16 days after altitude exposure, hemoglobin rates had already returned to their values in the PRE moment. With this, it is interesting to note that although greater hemoglobin rates are important to increase oxygen transport, such changes did not necessarily need to remain high for the athlete to achieve peak performance in tests with aerobic predominance, at least in the training model used. Gore et al.^14^ emphasize that improvements in performance after altitude training may be associated with a set of factors, not only related to hematological changes, but also greater muscle efficiency, probably at the mitochondrial level, angiogenesis, glucose availability, as well as greater clearance and ability to tolerate lactic acid production at sea level.

The analysis of centrality in complex networks sought to understand, through graphs, the integration of the studied variables before (PRE) and 15 days after altitude training of athletes at lower altitudes (POST2). We chose these two scenarios, considering that we observed, by conventional statistics, a significant reduction of time in the 3000 m (T3K) in POST2. As these athletes were mainly focused on long-distance and medium-distance running, the search for strategies such as the one investigated in our study (hypoxia–altitude training) is intended to have this better performance. We sought to study, through complex networks, how the different variables are integrated in the two scenarios, in order to understand, in terms of centrality (*pondered degree*, *pagerank*, and *betweenness*), how such relationships are established before and after the success with LHTH strategy. The graphs referring to *pondered degree* showed interesting characteristics before and 15 days after altitude training. In the PRE scenario, the graph presented slightly higher density than in the POST2 scenario (0.446 vs 0.418). Furthermore, the graph-based weighted Jaccard distance (dGWJ) showed that both networks PRE and POST2 are highly dissimilar, which corroborate the results summarized in Fig. 7. Moreover, in the POST2 scenario, the node-based weighted Jaccard distances (dNWJ) revealed that both variables Hb and Hct presented higher dissimilarity values from their node’s neighbors, compared with the PRE scenario. These results suggest to us that both variables have their discriminability properties increased in the POST2 scenario. In addition, there was a greater balance between the nodes in the positions of higher centralities (ranking) in the first scenario. After altitude training, graph analysis indicated higher ranking for parameters related to hematological characteristics, which was strongly evidenced for Hb and Hct, establishing that such nodes increased their connectivity with the others studied.

We also emphasize other physiological parameters clearly related to oxygen transport, which had their privileged positions in the POST2 graph—MCHC and RBC, for example. These responses to altitude reveal that, even for athletes with reduced capacity to adapt to training, due to the high performance already achieved, the effects of hypoxia in the LHTH model are quite effective. In relation to the *pondered degree*, in both scenarios, the T3K did not show much change in position between the training strategy adopted (13th and 11th). However, the minimum values of power, force and velocity, which were not different from the conventional statistics between the two moments, were presented in the analysis by complex networks, with significant evidence in the POST2 scenario, thus presenting a greater number of connections with other parameters. These nodes started in extremely low-ranking positions in the PRE scenario, and rose to incredible prominence in the POST2 graph. Considering these variables (HCHC and RBC) as of great importance for aerobic activities, the new higher-ranking positions of these nodes in the studied centralities (*pondered degree, pagerank* and *betweenness*) could mean that a greater interaction with the other analyzed parameters seems to be essential for high endurance performance. This brings understanding closer to justify the shortest times obtained for the 3000 m run after 15 days of return from training at altitude.

Quite interestingly, in terms of importance, all these vertices had similar design to the *pagerank* metric, suggesting that these first-grade parameters have more influence when compared to other parameters. This is in line with the strategy of high-altitude training, since in this metric the optimal placement of a node does not necessarily involve the number of connections, but rather with the connections with other centralizing nodes. For *pagerank*, the position of the T3K was the same in both scenarios (14th), suggesting equal integration between the other parameters that remained unchanged with the intervention at higher altitudes. This may suggest that, regarding performance, the relationship between nodes gives energy support to the task that, in fact, leads to gains in sports performance. Finally, despite Hb and Hct already appeared as important nodes in the intermediation between pair of nodes in PRE scenario, *betweenness* centrality analysis highlighted these nodes in POST2 condition, reinforcing their significance in an integrated way, potentiated by altitude training (Fig. 8, panel C). Thus, even with a consensus on superior endurance performance after training at altitude, our study showed that, for some aerobic parameters, there was no statistical significance (PRE versus POST2) when applied to the conventional analysis. However, especially for Hb and Hct, the analysis of complex networks reinforces these nodes as having the greatest centrality, which, despite showing minimal changes, are sufficient to promote the best performance in an integrative way.

## Conclusion

Our study confirms the positive effects of the LHTH model for high-performance long-distance Paralympic runners. Altitude can raise hematocrit and hemoglobin levels after 30 days of intervention, even for already highly trained athletes. After 5 days of readaptation, athletes showed a reduction in velocity, force and mechanical power in AO30, but after 16 days of readaptation at low altitudes, variables related to power and velocity returned to their initial conditions. Although losses did occur shortly after 5 days of readaptation, the performance in running 3000 m after 16 days of readaptation at low altitudes was high compared to the conditions that preceded altitude training, suggesting a clear beneficial effect of the LHTH model. Furthermore, the integrated model of complex networks confirms the prominence of hematological parameters, especially hematocrit and hemoglobin, as central nodes in this adaptive process. Thus, this study suggests that the LHTH model at an altitude of 2500 m for 30 days, followed by a period of 15–16 days of readaptation to low altitude, is effective in improving the physical performance of long- and medium-distance runners.

### Study limitations

Despite well conducted, our study shows some limitations. (1) In POST1 there is no hematological analysis, as it was performed on the last day of altitude and considering the resumption of training already focused on the next competitions, due to logistics, it was not possible to perform it after 5 days of returning from altitude; (2) despite the athletes were accompanied in the PRE, altitude and POST periods by nutritionists from the Brazilian Paralympic team, nutritional analysis was not conducted in the present study. In future opportunities, certainly we will follow this way; (3) the sample size was not to be higher because all Brazilian Paralympic athletes who perform tests in world competitions and Paralympics were studied. This choice of subjects was taken precisely for applications in complex networks, since due to the athletic conditions of these, we have a sample that is very focused on application in high performance; (4) in a way, the above limitation leads to the absence of a control group, as it is not possible to carry out a different treatment for the development of performance for athletes of this level. Thus, randomizing the study could hinder the sport development of this very specific sample of athletes. Future studies by our group will seek to minimize such limitations.
